# Readiness of Primary Healthcare Physicians for Providing Genetic Services

**DOI:** 10.7759/cureus.78031

**Published:** 2025-01-26

**Authors:** Abdulraheem A Almalki

**Affiliations:** 1 Department of Clinical Laboratory Sciences, College of Applied Medical Sciences, Taif University, Taif, SAU

**Keywords:** genetics, genomic services, prevention, primary health care, public health care

## Abstract

Background/objectives

The primary healthcare system plays an important role in addressing the health needs of communities and preventing diseases. The system in Saudi Arabia is undergoing a major transformation under Saudi Arabia’s Vision 2030, with the main aim of increasing the efficiency and accessibility of health services to all members of the community. This reform provides an opportunity to revolutionize patient care by integrating genetic services. This study assesses the readiness of primary healthcare physicians in Saudi Arabia to provide genetic services, identifies possible impacts and barriers to integrating genetic services, and examines the willingness of physicians to undergo further training if required.

Method

A cross-sectional study of 31 primary healthcare physicians was conducted between May 2024 and September 2024 using a self-structured questionnaire.

Results

Most primary healthcare physicians considered that they had insufficient formal education and limited knowledge of genetics; thus, they lacked confidence in providing genetic services to patients. They reported that several barriers to integration existed, including practitioners’ level of knowledge, the time required for each patient, the rarity of cases, and limited resources.

Conclusion

Despite the current knowledge gap, primary healthcare physicians recognized the value of genetic services and the need to integrate them into their practice and expressed interest in expanding their knowledge.

## Introduction

Primary healthcare is the first point of contact for individuals and families with the healthcare system. It provides accessible and affordable care for common and chronic illnesses and minor injuries, as well as preventive services. In Saudi Arabia, the primary healthcare system is structured around centers located in rural and urban areas throughout the country [1]. The system is currently undergoing a complete transformation as part of the country’s Vision 2030 initiative with the aim of improving its efficiency, quality, and accessibility. The central focus of the transformation is preventive care (prevention before treatment) to ensure that health issues are identified and managed early to create a healthier and more productive society [2].

The high rate of consanguinity in Saudi Arabia has been associated with the relatively common occurrence of population-specific autosomal recessive disorders. Novel genes linked to the Saudi population and the founder effect linked to certain tribes or populations exhibiting common disease-causing variants result in the need for well-established medical genetic services to meet the demand. Currently, these services are mainly provided at governmental tertiary hospitals and a few private laboratories and hospitals in large cities, with limited services available in rural areas [3]. Several programs have been initiated to reduce the incidence of genetic disorders. For example, a premarital screening program for hemoglobinopathies was initiated in 2004, which was accompanied by an awareness campaign [4]. More recently, the Saudi Genome Program was launched in 2018 as part of the broader Saudi Arabia’s Vision 2030 [2]. In addition, the Saudi Human Genome Program is a large-scale and government-funded initiative that aims to increase understanding of genetic disorders in Saudi Arabia, establish a national genetic database, and enhance preventive measures.

Advances in genetic research and the rapid development of genetic testing technologies have provided new insights into how genetics influence health and disease. The integration of genetic services into primary healthcare practices has the potential to revolutionize patient care by advancing precision medicine. Primary healthcare physicians are well positioned to incorporate genomic information into routine consultations, which can help them develop preventive strategies, increase the accuracy of assessments, facilitate early detection, and devise tailored treatment plans. However, such integration will require primary healthcare physicians to have up-to-date knowledge of modern genetics, as they will be expected to determine whether a patient or their family requires genetic investigation, request appropriate genetics tests, discuss management plans, refer patients to tertiary hospitals, and provide genetic counseling. Despite this potential, several studies have reported that genetic services are often regarded by general practitioners (GPs) as specialty services. Many GPs also lack the current knowledge and skills needed to incorporate modern genetic techniques into their practice [5,6].

The aims of this study are to assess the readiness of primary healthcare physicians in Saudi Arabia to provide genetic services, to identify possible impacts and barriers to integrating genetic services into primary healthcare, and to examine the willingness of physicians to undergo further training if required. The results will provide information about the current situation and inform future decisions related to the ongoing transformation plan.

## Materials and methods

Survey development, population, and data collection

A 22-question survey related to the objectives of the study was developed (Appendices). Survey development was an iterative process and guided by published work in this area [6-8]. Prior to administration, it was piloted on four primary healthcare physicians to evaluate the feasibility of the study design, readability of the items, and reliability of the questionnaire. Participants were asked to attend a meeting to fill out the survey and were then asked about what they thought each item and their response meant. Data from the pilot were included in the final analysis. 

The questionnaire was divided into four parts. The first part included four questions about the gender, title, years of experience, and workplace location of the participant. The second part included five questions that examined the participant’s current knowledge of genetics. The third part included eight questions that identified the current status of genetic services at the participant’s healthcare center. The fourth part included five questions that determined the perceived impact of providing genetic services and the barriers to their integration. All primary healthcare physicians practicing in Saudi Arabia were eligible to participate. 

The survey was web-based and had an average completion time of less than five minutes. It was conducted between May and September 2024. An invitation to complete the survey with a brief description of the study was shared through the head of a family medicine program in Makkah province. 

The study proposal (design, questionnaire, and informed consent) was reviewed and approved by the Taif University Ethical Committee (application no. 45-219). The survey was anonymous, and no personal information was collected.

Data analysis 

Descriptive statistics were used for each survey question, and results were presented using frequencies and percentages. The chi-squared test (χ2 test) was used to assess relationships between categorical variables. All data were imported, stored, and analyzed using Microsoft Excel (Microsoft® Corp., Redmond, WA).

## Results

Demographic factors

A total of 31 primary healthcare physicians agreed to complete the questionnaire. The majority of the participants were consultants (20 participants; 65%), with the remaining 11 participants comprising nine specialists and two GPs. The number of years of experience of the participants ranged between one and 13 years, with 10 years being the most common answer (11 participants). The participants were from the cities of Taif (12 participants), Makkah (11 participants), Riyadh (five participants), and Jeddah (three participants). 

Questionnaire results

For the five questions related to the current level of knowledge of genetics, the majority of participants gave negative responses (Table 1). Twenty-six participants (84%) stated that they had received no formal training or education in clinical genetics, 27 participants (87%) indicated that they were only slightly knowledgeable about clinical genetics, 13 participants (43%) stated that their current level of knowledge was inadequate to identify the signs of a possible genetic syndrome or disease, and 13 participants (43%) said that they are not sure. Twenty-three participants stated that their current level of knowledge is not adequate to order genetic tests, and only 16 participants (52%) believed that their current level of knowledge was sufficient to confidently refer a possible genetic syndrome or disease to a specialist. Based on the chi-squared test, there were no differences in the current level of knowledge between those who had no formal training and those who stated they had (p-value > 0.05 for all).

**Table 1:** Level of current knowledge

Questions	Options	Frequency	Percentage
Have you received any formal training or education in clinical genetics?	Yes	2	6%
	No	26	84%
	Not sure	3	10%
What is your current level of knowledge of clinical genetics practice?	Very knowledgeable	0	0%
	Moderately knowledgeable	3	10%
	Slightly knowledgeable	27	87%
	Not knowledgeable	1	3%
Is your current level of knowledge adequate to suspect a genetic syndrome/disease?	Yes	5	14%
	No	13	43%
	Not sure	13	43%
Is your current level of knowledge adequate to order genetic tests for your patients?	Yes	1	3%
	No	23	74%
	Not sure	7	23%
Is your current level of knowledge adequate to make a specialty referral for a suspicious case?	Yes	16	52%
	No	2	6%
	Not sure	13	42%

The next eight items in the questionnaire were used to assess the current status of genetic services at the participants’ healthcare center (Table 2). Three participants (10%) indicated that they never encountered medical genetic conditions in their practice, 23 participants (74%) indicated that they seldom encountered such conditions, and five participants (16%) indicated that they sometimes encountered medical genetic conditions. About half of the participants (15 participants; 48%) stated that they felt uncomfortable discussing genetic concepts with patients, and only five participants stated that they felt comfortable, with the remainder selecting the neutral option. Most participants (28 participants; 91%) indicated that they have never ordered genetic tests for their patients. Thirteen participants (43%) indicated that they lacked confidence in explaining the results of genetic tests to patients, 12 participants (39%) were slightly confident, one participant (3%) was reasonably confident, and three participants (10%) were extremely confident. Of the 31 participants, 19 (61%) indicated that they had never referred a patient to a specialist hospital owing to a suspected genetic disease. Twelve participants (39%) said that they had referred a patient but indicated that it was unusual for them to do so. 

**Table 2:** Status of current practice

Questions	Options	Frequency	Percentage
How often do you encounter medical genetic conditions in your practice?	Very often	0	0%
	Sometimes	5	16%
	Rarely	23	74%
	Never	3	10%
How comfortable do you feel discussing genetic concepts with patients?	Very uncomfortable	0	0%
	Uncomfortable	15	48%
	Neutral	11	35%
	Comfortable	5	16%
	Very comfortable	0	0%
Have you ever ordered genetic tests for your patients?	Yes	3	10%
	No	28	91%
How confident do you feel in interpreting genetic test results?	Extremely confident	3	10%
	Fairly confident	1	3%
	Slightly confident	12	39%
	Not confident	13	43%
Have you ever referred a patient to a genetic specialist?	Yes	12	39%
	No	19	61%
How often do you refer patients to a genetic specialist?	Very often	0	0%
	Sometimes	0	0%
	Rarely	12	39%
	Never	19	61%
Which of the following do you think physicians in primary healthcare are responsible for providing? (You can select more than one.)	Taking family history to identify a genetic condition	31	100%
	Assisting or counseling patients on genetic testing and results	26	84%
	Referring patients to specialists for advice and follow-up care	28	90%
	Warn families about risks in the family.	24	77%
Are you aware of the resources available, if any, to you for genetic consultation or support in your practice?	Yes	4	13%
	No	22	71%
	Not sure	5	16%

Participants were then given a list of services and asked which of the following they believed that primary care physicians had a responsibility to provide. The list comprised obtaining a family history to help identify a genetic condition, assisting or counseling patients on genetic testing and results, referring patients to specialists for advice and follow-up care, and warning families about possible genetic risks. All participants (100%) selected the first option, 26 participants (84%) selected the second option, 28 participants (91%) selected the third option, and 24 (78%) participants selected the fourth option. Participants were allowed to add other responsibilities, but no responses were given. They were then asked if they were aware of any resources available to them for genetic consultation or support in their practice. The majority (22 participants; 70%) were unaware of any resources, with only four participants (13%) stating that they knew of resources available and the remaining five participants stating that they were unsure whether resources were available.

The final five items in the questionnaire were used to identify any perceived impacts and barriers to the integration of genetic services at their healthcare center (Table 3). All participants (100%) indicated that incorporating genetic services into their practice would have a positive impact on patient care. In addition, nearly all participants (30 participants; 97%) indicated that incorporating genetic services into their practice would require additional resources. Participants were presented with four possible barriers to the incorporation of genetic services and a space to add any other perceived barriers. The four listed barriers were the practitioner’s level of knowledge, time required for each patient, rarity of cases, and required resources. Twenty-eight participants (90%) selected the first option, 22 participants (71%) selected the second option, 15 participants (48%) selected the third option, and 24 participants (77%) selected the fourth option. Most participants (26 participants; 84%) believed that incorporating genetic services into their practice would increase their job satisfaction as a primary care physician. Finally, 24 participants (77%) indicated that they were interested in expanding their knowledge and skills in clinical genetic services.

**Table 3:** Perceived impact and barriers

Questions	Options	Frequency	Percentage
Do you think incorporating genetic practice in your practice will have a positive impact on patient care?	Yes	100	100%
	No	0	0%
	Not sure	0	0%
Do you think incorporating genetic practice in your practice requires additional resources?	Yes	30	97%
	No	0	0%
	Not sure	1	3%
Which of the following do you think will be a barrier to incorporating genetics practice in your practice? (You can select more than one.)	Practitioner level of knowledge	28	90%
	Time required for each patient	22	71%
	Rarity of cases	15	48%
	Resources	24	77%
Do you think incorporating genetics practice in your practice will increase your job satisfaction as a primary care physician?	Yes	26	84%
	No	2	6%
	Not sure	3	10%
How interested are you in expanding your knowledge and skills in clinical genetics practice?	Interested	24	77%
	Not interested	7	23%
	Not sure	0	0%

## Discussion

Most participants reported that they had no formal training or education in clinical genetics and admitted to being only slightly knowledgeable about clinical genetics. Nearly half the participants thought that their knowledge was inadequate to identify the signs of a possible genetic condition, and most of the remaining participants were uncertain. Only 52% of participants believed that they had sufficient knowledge to refer patients for specialized care. Practitioners’ limited knowledge, lack of confidence, and rarity of referrals have been reported elsewhere [6,9,10]. 

The primary healthcare physicians believed that they were not yet ready to integrate genetic services into their practice due to their lack of formal education and training. This skills gap can result in patients with a genetic disorder or at risk of developing one being undiagnosed, which may lead to complications. Some genetic disorders require early attention to manage the symptoms and prevent progression. Patients with such a condition may not currently receive such attention. In a population with a high rate of consanguinity, such as that in Saudi Arabia, early risk assessment and family awareness are critical to limit the impact of diseases and prevent the transmission of certain conditions [3]. Although half of the participants believed that they had sufficient knowledge to refer patients for specialized care, referrals were most likely due to uncertainty about symptoms and made without functional data. This was due to physicians’ lack of formal training and limited knowledge of clinical genetics, which may lead to delays in patients receiving an accurate diagnosis. 

The participants generally had few encounters with genetic conditions (74% rarely, 16% sometimes). They also admitted their discomfort in discussing genetic concepts with patients (48%), their low confidence in ordering or interpreting genetic tests, and their rare referrals to specialists. Such limited exposure of primary healthcare physicians has been a common observation in other studies [11-14]. This may reflect the lack of knowledge and training in clinical genetics, which is hindering physicians from recognizing genetic conditions rather than the rarity of diseases. The discomfort and low confidence of the participants are also considered to be related to their lack of education in genetics, which could discourage their active integration of genetic services into their practice. 

Nearly all participants acknowledged the importance of their role in obtaining a family history, patient counseling, referrals, and risk communication, and all participants considered that the integration of genetic services would positively impact patient care. Similar views of practitioners on obtaining a family history and performing tasks such as counseling, referrals, and risk communication have been reported in several studies [14-17]. Although some practitioners believe that these tasks are part of their role, others remain skeptical [5,18-20]. However, practitioners’ lack of formal education in genetics and limited resources may reduce their ability to implement genetic services.

In addition to their limited education and resources, participants believed that the time required for patient consultations and the rarity of cases may be barriers to implementing genetic services (71% and 48%, respectively). Time constraints have been previously reported to be a barrier by GPs [9,21]. Despite their acknowledgment of several barriers, participants showed a strong interest in expanding their knowledge and skills in genetics (78%), in agreement with several previous studies [22-26]. This finding emphasizes the need for targeted interventions that address barriers preventing physicians from adopting genetic services as part of their routine work. Successful clinical interventions that improved practitioners’ knowledge and confidence have been reported. For example, Carroll et al. reported that a multifaceted knowledge translation intervention to increase practitioners’ knowledge that included a practical tools portfolio and interactive workshop significantly enhanced practice and confidence among family physicians [27]. In addition, Houwink et al. indicated that an online development module about oncogenetics increased the knowledge of practitioners, with participants reporting high satisfaction with the module [28]. 

Note that there were no significant differences in the responses between the 28 participants who had received no formal training or education in genetics and the three participants who had received formal training or education. In addition, title, years of experience, and workplace location had no significant impact on participants’ responses.

This study was conducted to explore current practices and barriers. There are potential limitations to consider when interpreting the findings. Firstly, the study had a low response rate, and most participants were from Makkah province; thus, the results may not be generalizable. Secondly, the information provided was self-reported, which might be prone to recall or desirability biases. However, the fact that the survey was anonymous and no personal information was collected may suggest that the responses were likely authentic. Further studies with larger sample sizes and the inclusion of other provinces are needed for a more comprehensive understanding. Incorporating a focus group approach is also recommended to accomplish this.

## Conclusions

With the rapid advances in clinical genetics, there is significant potential to transform patient care from traditional approaches to personalized or precision medicine by integrating genetic services into primary healthcare at the earliest stages. Such a transformation has the potential to enhance the efficiency, quality, and accessibility of healthcare services in alignment with Saudi Arabia’s Vision 2030 and its ongoing healthcare reform initiatives. Such integration would require prioritizing education and training in genetics for healthcare professionals working in primary care settings. A targeted, continuous educational program, in which the GPs in this study expressed interest, could address the current knowledge gap. Other anticipated barriers, such as time constraints and resource limitations, must also be addressed to ensure the successful implementation of genetic services into primary healthcare.

## Appendices

A.   Demographics 

1.     Gender

·      Male

·      Female

2.     Title

·      General practitioner

·      Resident

·      Specialist

·      Consultant

3.     Years of working experience 

………………………………………………

4.     Workplace location (city)

………………………………………………

B.    Level of current knowledge 

1.     Have you received any formal training or education in clinical genetics? 

·      Yes

·      No

·      Not sure

2.     What is your current level of knowledge of clinical genetics practice?

·      Very knowledgeable

·       Moderately

·       Slightly/not sure

3.     Is your current level of knowledge adequate to suspect a genetic syndrome/disease? 

·      Yes

·       No

·       Not sure

4.     Is your current level of knowledge adequate to order genetic tests for your patients?

·      Yes

·      No

·      Not sure

5.     Is your current level of knowledge adequate to make a specialty referral for a suspicious case?

·      Yes

·      No

·      Not sure

C.   Status of current practice

1.     How often do you encounter medical genetic conditions in your practice?

·      Very often

·       Sometimes

·       Rarely

·       Never

2.     How comfortable do you feel discussing genetic concepts with patients?

·      Very uncomfortable

·      Uncomfortable

·      Neutral

·      Comfortable

·      Very comfortable

3.     Have you ever ordered genetic tests for your patients?

·      Yes

·      No

4.     How confident do you feel in interpreting genetic test results?

·      Extremely confident

·      Fairly

·      Slightly

·      Not confident 

5.     Have you ever referred a patient to a specialist hospital for a suspicion of a genetic disease?

·      Yes

·      No

6.     How often do you refer patients to a specialist hospital for a suspicion of a genetic disease?

·      Very often

·       Sometimes

·       Rarely

·       Never

7.     Which of the following do you think primary care physicians are responsible for providing? (You can select more than one.)

·      Taking family history to identify a genetic condition

·      Assisting or counseling patients on genetic testing and results

·      Referring patients to specialists for advice and follow-up care

·      Warning families about risks in the family.

              Others…………………….

8.     Are you aware of the resources available, if any, to you for genetic consultation or support in your practice?

·      Yes

·       No

·       Not sure

D.   Perceived impact and barriers

1.     Do you think incorporating genetic practice in your practice will have a positive impact on patient care?

·      Yes

·      No

·      Not sure

2.     Do you think incorporating genetic practice in your practice requires additional resources?

·      Yes

·       No

·       Not sure

3.     Which of the following do you think will be a barrier to incorporate genetics practice in your practice? (you can select more than one):

·      Practitioner level of knowledge

·      Time required for each patient

·      Rarity of cases

·      Resources 

Others……………..

4.     Do you think incorporating genetics practice in your practice will increase your job satisfaction as a primary care physician?

·      Yes

·       No

·       Not sure

5.     How interested are you in expanding your knowledge and skills in clinical genetics practice?

·      Interested

·      Not interested

·      Not sure
